# Assessment of the Aromatase Inhibitory Activity of Ma-Huang-Tang (MHT) and Its Active Compounds

**DOI:** 10.1155/2019/4809846

**Published:** 2019-12-18

**Authors:** Dong Ho Jung, Joo Tae Hwang, Bo-Jeong Pyun, Song Yi Yu, Byoung Seob Ko

**Affiliations:** Country Herbal Medicine Research Division, Korea Institute of Oriental Medicine, Daejeon 34054, Republic of Korea

## Abstract

Aromatase, a cytochrome P450 enzyme that converts androgens into estrogens, is an important drug target for hormone-dependent diseases. The purpose of this study was to elucidate the aromatase inhibitory effects of Ma-Huang-Tang (MHT), a traditional Korean herbal medicine prescription, and to identify its active ingredients. In this study, the inhibitory effect of MHT on aromatase activity was observed using dibenzylfluorescein (DBF) and KGN cells, and the dose-dependent effect of MHT was verified (IC_50_ values of 251 *μ*g/mL and 246 *μ*g/mL as determined by the two methods, respectively). Furthermore, among the six herbal medicines that constitute MHT, *Ephedrae Herba*, *Cinnamomi Ramulus*, and *Glycyrrhizae Radix et Rhizoma* showed the most potent inhibition of aromatase activity. Furthermore, upon identification of the active MHT compounds, three markers from *Glycyrrhizae Radix et Rhizoma*, liquiritin (5), liquiritin apioside (6), and liquiritigenin (7), were verified (IC_50_ values of 530 *μ*M, 508 *μ*M, and 1.611 mM and 499 *μ*M, 522 *μ*M, and 1.41 mM as determined by the two methods, respectively). In addition, their contents were confirmed to be 15.58, 19.80, and 2.22 mg/g, respectively, by HPLC/DAD analysis. These results indicate that the aromatase inhibitory effect of MHT results from the synergistic action of its active components and that MHT has potential as a preventive agent against aromatase activity.

## 1. Introduction

Aromatase (CYP19) is a major cytochrome P450 (CYP) steroidogenic enzyme that catalyzes the rate-limiting aromatization step of converting androgens, including testosterone and androstenedione, into estrogens such as estrone and 17*β*-estradiol [1, 2]. Aromatase enzymatically removes C19 using C19 androgens as substrates and forms a phenolic A ring in the steroid [3, 4]. Through these actions, the androgen/estrogen ratio is controlled to maintain the endocrine balance. Therefore, the regulation of aromatase expression is important because aromatase is a vital target of drugs used to treat hormone-dependent diseases, including breast cancer, prostate cancer, and other diseases associated with aromatase overexpression, such as hormone disorders and premature ejaculation [5–7].

The main uses of aromatase inhibitors (AIs) include preventing the recurrence of estrogen-specific receptor-positive breast cancer and reducing the risk of adjuvant treatment [8]. Therefore, the use of AIs to reduce estrogen production can be a suitable approach for treating related tumors [9]. Even in men, several studies have yielded positive results using AIs. For example, improvements in hormone profiles induced by AIs have been identified in men suffering from infertility and low testosterone [10, 11]. In addition, positive effects of AIs on cognition were reported [12], and AIs were reported to be effective in treating male breast cancer [13].

Previous studies have proposed a variety of AI screening techniques based on a cell-free assay using human placental microsomes and human recombinant aromatase proteins and a cell-based assay using a human granulosa-like tumor cell line (KGN cells) [14–16]. Dibenzylfluorescein (DBF) is broadly used as a profluorescent probe substrate in high-throughput assays (HTS), such as those for CYP1A2, CYP2B6, CYP2C19, CYP3A4, and CYP19 [17–19]. DBF can be easily applied in development curve analysis and is suitable for initial screenings to identify compounds that require additional investigation in optimizing drug interactions [20]. In addition, cell-based aromatase activity assays have been established and developed in a variety of cells. Among them, a method using KGN cells, a steroid-producing human ovarian granular tumor cell line established from patients with invasive granulosa cell cancer, has been reported [21]. KGN cells are characterized by their inability to synthesize androgen or estrogen on their own due to their lack or low levels of 17*α*-hydroxylase. Therefore, the determination of aromatase activity can be an efficient evaluation method based on the simple cultivation of cells with androstenedione and the subsequent measurement of the estrone level in culture medium via an enzyme-linked immunosorbent assay (ELISA). In addition, the cellular toxicity of the drug materials being assessed can be determined [16].

Traditional alternative medicine has the advantages of subject diversity and multiple active ingredients, and the use of alternative medicines, such as herbal medicines, which are known to contain active medicinal ingredients that have physiological effects on health, has increased [22, 23]. Ma-Huang-Tang (MHT), an oriental herbal formula that has been used in Korea for a long time, is composed of six herbs, *Ephedrae Herba*, *Cinnamomi Ramulus*, *Glycyrrhizae Radix et Rhizoma*, *Armeniacae Semen*, *Zingiberis Rhizoma Recens*, and *Allii Radix*, and has traditionally been used in the treatment of sweating, asthma, febrile disease, and inflammatory skin diseases [24–26]. MHT also reportedly improves steroid-induced gene expression and ovarian dysfunction [27]. In this regard, the exact mechanism of MHT has not been revealed, and there are no reports on the effects of MHT on aromatase activity, which could affect the maintenance of hormonal balance. Therefore, we investigated the inhibitory effect of aromatase activity on MHT and its active compounds using cell-free and cell-based *in vitro* assays and established a reproducible analysis method for identifying active compounds using high-performance liquid chromatography with diode-array detection (HPLC-DAD).

## 2. Materials and Methods

### 2.1. General Material

Androstenedione, DBF, dimethyl sulfoxide (DMSO), glucose-6-phosphate, glucose-6-phosphate dehydrogenase, NADP^+^, MgCl_2,_ albumin, letrozole, chrysin, and potassium phosphate were obtained from Sigma-Aldrich (St. Louis, MO, USA). Supersomes human CYP19 was obtained from Corning (Woburn, MA, USA). Analytical-grade acetonitrile, methanol, and ultrapure water were purchased from J. T. Baker (Phillipsburg, NJ, USA). Trifluoroacetic acid was of analytical grade and was purchased from Sigma-Aldrich.

### 2.2. Standards and Solutions

Standards of ephedrine hydrochloride were obtained from the MFDS (Ministry of Food and Drug Safety), and coumarin, cinnamic acid, cinnamaldehyde, liquiritin, liquiritin apioside, liquiritigenin, and glycyrrhizin were purchased from Sigma-Aldrich (St. Louis, MO, USA). Stock solutions of four chemicals (ephedrine hydrochloride, liquiritin, liquiritin apioside, and liquiritigenin) were prepared at a concentration of 1 mg/mL in methanol. The mixed standard working solutions were diluted with distilled water to a final concentration of 0.0001 mg/mL. The working solutions were stored at +4°C prior to analysis.

### 2.3. Preparation of MHT and Its Components

The samples of MHT and its six herbal components (2012-KE47-1∼2012-KE47-6) were validated by the K-herb Research Group of the Korea Institute of Oriental Medicine.

### 2.4. Cell Culture and Treatment

The human granulosa-like tumor cell line KGN was obtained from RIKEN BioResource Center (Ibaraki, JPN). The cells were grown in Dulbecco's modified Eagle's medium/Ham's F-12 nutrient mix (DMEM/F-12, Welgen, Daejeon, KOR) supplemented with 5% (v/v) fetal bovine serum (FBS) and 0.5% antibiotics (100 U/mL penicillin and 100 *μ*g/mL streptomycin, Gibco BRL, NY, USA) in a humidified atmosphere of 5% CO_2_ at 37°C. Prior to treatment with MHT and its components in all the experiments, the cells were cultured for 24 h by switching to phenol-red free DMEM/H-12 medium (Welgen, Daejeon, KOR) with 5% (v/v) charcoal-stripped FBS (HyClone, Logan, UT, USA), and the samples were treated under the same conditions.

### 2.5. Aromatase Inhibition Assay

The inhibition of aromatase activity by MHT and each component was assessed by measuring the fluorescence intensity of fluorescein produced by the hydrolysis of DBF [20, 28]. The 10 *μ*L samples of MHT at each concentration and of the MHT components were preincubated at 37°C for 30 min with 90 *μ*L of NADPH regenerative buffer (2.6 mM NADP^+^, 7.6 mM glucose-6-phosphate, 0.8 U/mL glucose-6-phosphate dehydrogenase, 13.9 mM MgCl_2_, and 1 mg/mL albumin in 50 mM potassium phosphate, pH 7.4). A 100 *μ*L mixture of aromatase and substrate (2 *μ*M DBF, 40 pmol/mL aromatase, and 4 mg/mL albumin in 50 mM potassium phosphate, pH 7.4) was then added to the sample, and the resulting mixture was incubated for 30 min at 37°C in the dark. After the reaction, the sample was incubated with 150 *μ*L of 2N NaOH for an additional 2 h at 37°C to remove impurities other than fluorescein. Subsequently, the fluorescence of the sample was measured using a Synergy/HTX multimode reader (Bio-Tek Instruments Inc., VT, USA) with excitation at 485 nm and emission at 535 nm. All experiments were performed three times at separate times.

### 2.6. ELISA of Estrone

Cells (4 × 10^4^ cells/mL) were placed on a 96-well tissue culture plate and incubated in DMEM/F-12 medium with 5% charcoal-stripped FBS (HyClone, Logan, UT, USA) overnight. After the addition of MHT or its components, the cells were incubated for 24 h. Subsequently, 0.1 mM androstenedione was added to each well, and the cells were incubated for 24 h. Fractions of the supernatant were harvested and transferred to a new Eppendorf tube. The estrone concentration in each of the samples was measured at 450 nm using a Synergy/HTX multimode reader (Bio-Tek Instruments Inc., VT, USA) with an estrone ELISA kit (Abnova, CA, USA), and the cell viability was measured using a CCK-8 assay (Dojindo Laboratories, Tokyo, JPN).

### 2.7. Chromatographic Conditions for HPLC Validation

The HPLC analysis was conducted with a Shimadzu LC-20A Prominence Series system (Shimadzu Corporation, Kyoto, Japan) equipped with a quaternary pump (LC-20AD), vacuum degasser (DGU-20A3R), autosampler (SIL-20A), column oven (CTO-20A), and photodiode-array detector (SPD-M20A). The chromatographic data were interpreted using LabSolutions Multi-PDA software. Chromatographic separation was performed on a YMC Triart C18 column (4.6 × 250 mm i.d., 5 *μ*m). The column oven was maintained at 40°C, the detection was conducted at *λ* = 207 nm, and online UV absorption spectra were recorded in the range of 190 to 400 nm. The gradient elution was performed under the following conditions: initial mobile phase, acetonitrile/0.1% trifluoroacetic acid in water = 15 : 75 (*v*/*v*), 0∼15 min; 15 : 75 to 35 : 65 (*v*/*v*), 0∼10 min; 35 : 75 to 50 : 50 (*v*/*v*), 10∼20 min; 50 : 50 to 100 : 0 (*v*/*v*), 20∼25 min; and 100 : 0 (*v*/*v*), 25∼30 min. The flow rate was 1.0 mL/min, and the injection volume was 5 *μ*L.

### 2.8. Sample Preparation for HPLC

After transferring the dried MHT powder (5 mg) to a 20 mL flask, 10 mL of water was added and extracted under sonication for 10 min. After centrifugation for 10 min at 4,000 g, the supernatant was filtered with a disposable syringe filter unit (0.22 *μ*m, 25 mm, CA syringe filter) purchased from Futecs Co., LTD. (Daejeon, Korea) prior to injection into the HPLC system.

### 2.9. Method Validation for HPLC

The developed HPLC analysis method was validated according to linearity, accuracy, precision, limit of detection (LOD), and limit of quantification (LOQ) values. Based on guidance from the International Conference on Harmonization (ICH), the linearity was tested by calculating the value of *r*^2^ (correlation coefficient) for a calibration curve comprising eleven serial concentrations, and the precision of the developed method was assessed via the intermediate evaluation method using measurements of intra- and interday variability. The relative standard deviation (RSD) was considered a measure of the method precision, and recovery tests were performed to estimate the accuracy. The recovery was estimated by comparing the mean recovery (%) of the standards from the spiked extract and nonspiked extract samples. The LOD and LOQ were measured by the signal-to-noise ratio (S/N) method.

### 2.10. Statistical Analysis

The results are expressed as the means ± SEMs from multiple experiments. Paired Student's *t*-tests with Tukey's multiple comparison tests were performed to compare two groups using PRISM software (GraphPad, CA, USA). A *p* value <0.05 was considered to be statistically significant. Additionally, IC_50_ values were calculated by sigmoidal dose-response curve fitting (variable slope) using PRISM software.

## 3. Results

### 3.1. Inhibitory Effect of MHT Hydrothermal Extract on Aromatase Activity

**Figure 1:**
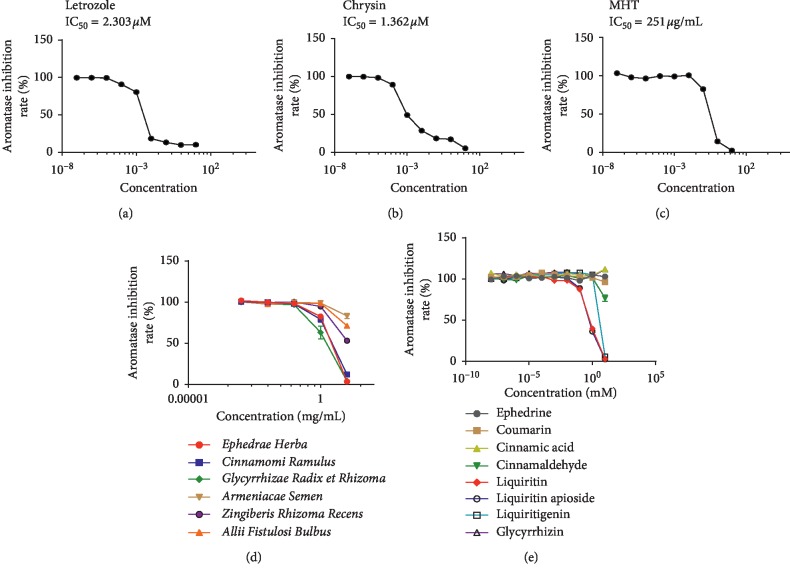
Inhibitory effects of letrozole (a), chrysin (b), MHT hydrothermal extract (c), the compositional herbs of MHT hydrothermal extract (d), and the major compounds of MHT hydrothermal extract (e) on aromatase activity as determined in a cell-free system.

The effect of MHT hydrothermal extract on aromatase activity was investigated by an *in vitro* fluorescence-based aromatase assay. As shown in Figure 1(c), MHT significantly reduced the aromatase enzyme activity during DBF hydrolysis in a dose-dependent manner, and the IC_50_ value was determined to be 251 *μ*g/mL. To ensure the accuracy of the experiment, letrozole and chrysin were used as comparative control groups, and they were also found to inhibit aromatase activity in a dose-dependent manner with IC_50_ values of 2.303 *μ*M and 1.362 *μ*M, respectively (Figures 1(a) and 1(b)). These results demonstrate that MHT inhibits the activity of aromatase.

### 3.2. Inhibitory Effects of MHT Hydrothermal Extract Components on Aromatase Activity

We examined the aromatase inhibitory effects of the six herbal medicine components of MHT hydrothermal extract: *Ephedrae Herba*, *Cinnamomi Ramulus*, *Glycyrrhizae Radix et Rhizoma*, *Armeniacae Semen*, *Zingiberis Rhizoma Recens*, and *Allii Radix*. As shown in Figure 1(d), aromatase assays were performed at six concentrations (0.0001∼10 mg/mL), and the IC_50_ values of *Ephedrae Herba*, *Cinnamomi Ramulus*, and *Glycyrrhizae Radix et Rhizoma* were 1.465, 1.535, and 1.247 mg/mL, respectively (Table 1). In contrast, the IC_50_ values of the other herbal medicines (*Armeniacae Semen*, *Zingiberis Rhizoma Recens*, and *Allii Radix*) were greater than 10 mg/mL. These results demonstrate that *Ephedrae Herba*, *Cinnamomi Ramulus*, and *Glycyrrhizae Radix et Rhizoma* are the most potent AIs among the components of MHT hydrothermal extract.

### 3.3. Inhibitory Effects of Major MHT Hydrothermal Extract Compounds on Aromatase Activity

**Figure 2:**
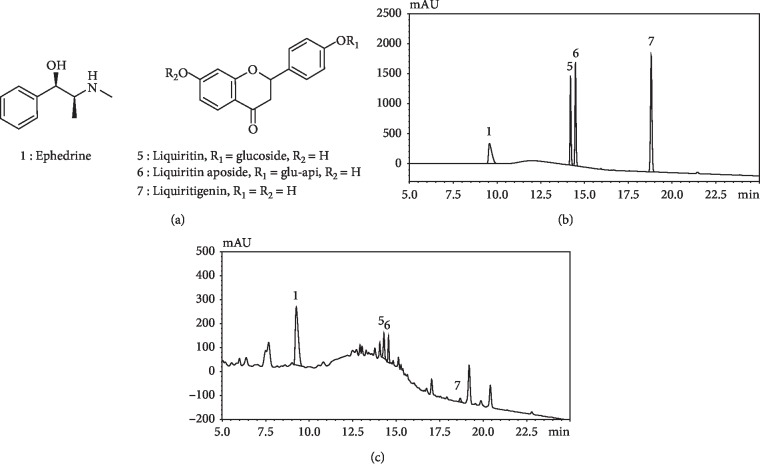
The HPLC analysis data of MHT extract: (a) The structures of four compounds. (b) The HPLC chromatograms of the standard mixture 0.2 mg/mL, *λ* = 207, of ephedrine (1) at 9.65 min, liquiritin (5) at 14.20 min, liquiritin apioside (6) at 14.49 min, and liquiritigenin (7) at 18.81 min. (c) The extracted MHT sample (0.5 mg/mL).

**Figure 3:**
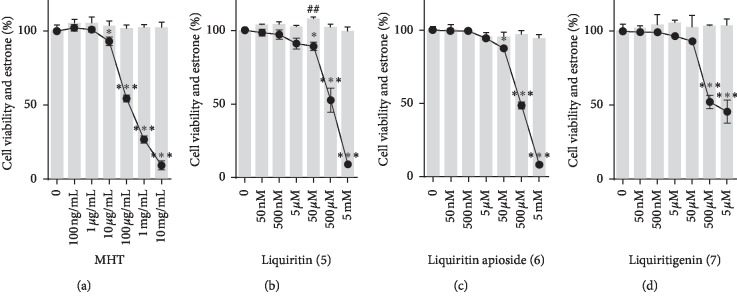
Inhibitory effects of three active compounds of MHT hydrothermal extract, liquiritin (5), liquiritin apioside (6), and liquiritigenin (7), on aromatase activity as determined using a cell system. Data represent the means ± SEMs of three experiments. (a)^*∗*^*p* < 0.05, ^*∗∗∗*^*p* < 0.05 vs. the nontreated group. (b)^*∗*^*p* < 0.05, ^*∗∗∗*^*p* < 0.05 vs. the nontreated group, ^##^*p* < 0.05 vs. the nontreated group. (c)^*∗*^*p* < 0.05, ^*∗∗∗*^*p* < 0.05 vs. the nontreated group. (d)^*∗∗∗*^*p* < 0.05 vs. the nontreated group.

We next investigated the effects of eight typical index compounds from the three MHT hydrothermal extract herbal medicine components identified as inhibiting aromatase activity: ephedrine (1) from *Ephedrae Herba* [29]; coumarin (2), cinnamic acid (3), and cinnamaldehyde (4) from *Cinnamomi Ramulus* [30]; and liquiritin (5), liquiritin apioside (6), liquiritigenin (7), and glycyrrhizin (8) from *Glycyrrhizae Radix et Rhizoma* [31]. As shown in Figure 2, the inhibition of aromatase activity was expressed as the percentage of fluorescein activity for each compound at concentrations ranging from 10 pM to 10 mM. The IC_50_ values of liquiritin (5), liquiritin apioside (6), and liquiritigenin (7) were 530* μ*M, 508 *μ*M, and 1.611 mM, respectively (Table 2). However, IC_50_ values greater than 10 mM were observed for the other main compounds (ephedrine (1), coumarin (2), cinnamic acid (3), cinnamaldehyde (4), and glycyrrhizin (8)). As shown in Figure 3, aromatase inhibition assays were performed by measuring estrone levels in KGN cells treated with MHT and the three major compounds liquiritin (5), liquiritin apioside (6), and liquiritigenin (7). Estrogen levels were decreased in a dose-dependent manner in MHT-treated KGN cells under conditions that did not affect their survival, and the IC_50_ value of MHT was confirmed to be 246 *μ*g/mL. Similarly, the IC_50_ values of liquiritin (5), liquiritin apioside (6), and liquiritigenin (7), the three major compounds in MHT, were determined to be 499 *μ*M, 522 *μ*M, and 1.41 mM, respectively (Table 3). Taken together, these results demonstrate that MHT and its main compounds exhibited IC_50_ values in the cell-based assay similar to those observed in the fluorescence assays using human recombinant microsomes and showed an inhibitory effect on aromatase.

### 3.4. Optimization of Chromatographic Conditions

As mentioned above, we identified the three main compounds responsible for the estrogen-reducing activity of MHT. Therefore, to investigate the contents of the active MHT compounds, an accurate, fast, and reproducible analytical method was carried out using HPLC/DAD by modifying conditions previously reported in studies that conducted quantitative analysis of 12 index compounds of MHT [31]. This study focused on developing the most efficient analytical method by targeting ephedrine (1), which is the major component originating from *Ephedrae Herba*, and liquiritin (5), liquiritin apioside (6), and liquiritigenin (7) derived from *Glycyrrhizae Radix et Rhizoma*, which were determined to be the active components of MHT. For efficient separation and detection of the four selected compounds, a gradient mixture of distilled water containing 0.1% trifluoroacetic acid and acetonitrile was used and was found to be the most suitable. The wavelength for detection was determined by comparing and analyzing the maximum UV absorbance profiles of each compound, and 207 nm was selected as the optimal single wavelength at which all four compounds could be detected. For the analytical column, we used a Triart C18 ExRS resin, which is relatively more hydrophobic than a normal C18 resin, making it easier to separate compounds with smaller structural differences. As a result, the similar structures of two of the active compounds evaluated, liquiritin (5) and liquiritin apioside (6), were analyzed with high resolution. Under these HPLC conditions, all compounds were free of interference from any other components and showed retention times of 9.65 (1), 14.20 (5), 14.49 (6), and 18.81 (7) min, respectively. The contents of the four compounds were successfully determined to be 135.80 mg/g for 1, 19.80 mg/g for 6, 15.58 mg/g for 5, and 2.22 mg/g for 7 (Figure 2 and Table 4).

### 3.5. Validation of the Developed Analytical Method

The linearity of the method was evaluated at eleven total concentrations ranging from 0.0001 to 0.4 mg/mL with five repetitions for each compound. The individual calibration curves were proven to have good linearity, as the correlation coefficient values (*r*^2^) were above 0.9999 (Table 1).

To assess the precision of the method, the following RSD values of the analyses, measured on the same day, were determined: 0.13∼0.89% for 1, 0.06∼0.83% for 5, 0.04∼0.68 for 6, and 0.12∼2.06 for 7. The interday precision was determined to be 0.25∼0.59% for 1, 0.18∼0.40% for 5, 0.05∼0.39% for 6, and 0.07∼0.18% for 7. Additionally, to confirm the accuracy of the analytical method, a recovery test was conducted by spiking standard solutions that were prepared at three different concentrations. The obtained recovery rates ranged from 100.2∼102.9% for 1, 99.6∼101.8% for 5, 97.8∼101.0% for 6, and 101.8∼102.4% for 7. Thus, the abovementioned accuracy and precision test results proved that the analyses of the four compounds contained in the MHT extract are reliable and reproducible (Table 5).

LOD and LOQ values were calculated by applying the SD values (*n* = 5) for regression analysis of five low concentrations (0.0001 to 0.001 mg/mL). The LOD and LOQ values were calculated to be less than 0.5774 and 1.7497 *μ*g/mL, respectively, which were lower than those of the tested compounds in the MHT extract (Table 6).

## 4. Discussion

In this study, we demonstrated for the first time the potential of MHT as an effective inhibitor of aromatase through cross-examination using a cell-free screening system and KGN cells. Furthermore, aromatase inhibitory activity was assessed for each of the compounds isolated from the MHT components, among which liquiritin (5), liquiritin apioside (6), and liquiritigenin (7) were demonstrated to be active. To date, various studies on methods for analyzing MHT components have been reported [31, 32]. As an example, prescription drugs may vary slightly in composition and proportion depending on the patient's condition. Therefore, a recent study examined these compositions and inferred the optimal prescription conditions using structural demand modeling (SEM), including HPLC analysis and the active correlation of the components [29]. However, most studies made efforts to most accurately detect the components that represent all the drugs that comprise MHT apart from its biological activity. In fact, the development of an analytical method that can detect multiple components is very important in terms of prescription drug quality control, as these drugs contain various medicinal materials in combination. However, in the process of evaluating the new activity, as reported herein, it is also very important to identify the effective active components of the prescription drug and to examine their contents to determine how much these components truly contribute to the biological activity of the drug. Therefore, this study conducted a validation protocol, including quantitative analysis, to investigate the practical effects of the newly identified MHT compounds with aromatase inhibitory activity.

Aromatase, a member of the cytochrome P450 enzyme family that is encoded by a single gene, CYP19A1, and controlled by a specific promoter, is expressed in various tissues, including the ovaries, placenta, bone, brine, skin, and adipose tissue [33]. AIs are classified as steroidal or nonsteroidal; type I (steroidal) AIs inhibit enzyme activity by irreversibly binding aromatase, and type II (nonsteroidal) AIs inhibit enzyme activity by reversibly binding enzyme substrates [34]. Retrozole, a nonsteroidal triazole derivative, and chrysin, a natural flavonoid present in plants, are among these representative AIs. Chlebowski et al. and Bao et al. reported that compared to other treatments, AIs are effective at increasing the survival rate of patients. AIs have also been demonstrated to have toxicity within an acceptable range [35, 36] To date, improved synthetic AIs have shown better side effect profiles than conventional AIs such as tamoxifen, but serious side effects of these drugs are still reported, such as those related to the bones, brain, and heart [37–43]. Many natural products or drugs can potentially be good candidates for use as chemicals in terms of safety because of their low toxicity [44–47]. Therefore, many natural products that have traditionally been used for medicinal purposes can also serve as AIs with reduced side effects [48, 49].

MHT has traditionally been used in the treatment of diseases such as influenza-like illnesses, sweating, asthma, fever, and female-related disorders [27, 50, 51]. However, the detailed mechanism of MHT in female-related disorders has not yet been clarified. Functionally, aromatase plays a key role in the irreversible conversion of androstenedione and testosterone into estrone and estradiol. Therefore, it is very important for maintaining the balance of endocrine hormones and is becoming a major target of drug development to treat diseases related to hormonal or aromatase overexpression, such as breast cancer, prostate cancer, growth disorder, and sexual precocity [7]. However, the aromatase inhibitory activity of MHT has not been reported to date.

In the present study, the inhibitory effect of MHT hydrothermal extract on aromatase activity was evaluated with a cell-free assay system based on DBF. The IC_50_ values of chrysin and retrozole were compared as positive controls, which confirmed that they were potent AIs. However, the disadvantage of these methods is that they cannot verify the toxicity and intracellular fate of the compounds being assessed; thus, we supplemented these methods with a method using KGN cells. The amount of estrone converted in KGN cells was dose-dependently decreased by MHT, and no cytotoxicity of MHT was observed. Furthermore, the aromatase inhibitory activity of the six herbal medicines that make up MHT was assessed and was confirmed in three of them: *Ephedrae Herba*, *Cinnamomi Ramulus*, and *Glycyrrhizae Radix et Rhizoma*. Based on these results, an additional experiment was conducted to identify the active ingredients of these three medicinal herbs that make up MHT, and eight compounds were selected according to the previously reported literature. Among the candidate compounds, effects of liquiritin (5), liquiritin apioside (6), and liquiritigenin (7) were observed, whereas all of the remaining compounds showed IC_50_ values above 10 mM. Among the three active compounds, liquiritigenin, a flavanone in which the sugar moiety has been removed from liquiritin and liquiritin apioside, is already well known as the major bioactive component of *Glycyrrhizae Radix et Rhizoma*. In particular, molecular modeling/docking studies on liquiritigenin have shown that it can form stable complexes with aromatase, and the selective estrogen receptor modulator (SERM) effect and relevance of the estrogen receptor (ER) modulating elements CECR6, NKG2E, and NKD in MCF-7 human breast cancer cells have been reported [52]. On the other hand, ephedrine, derived from *Ephedrae Herba*, an important material that forms MHT, was determined to be a major ingredient in this experiment but was not an important factor that inhibited aromatase. Overall, the results of this experiment suggest that the inhibitory activity of MHT on aromatase activity is derived from synergistic actions of the component drugs and compounds. Therefore, we plan to comprehensively interpret the above results and conduct further experiments to control the amount of *Glycyrrhizae Radix et Rhizoma,* the herbal medicine containing the active ingredients, contained in MHT and to thus create a more active prescription.

## 5. Conclusions

In conclusion, we report for the first time that MHT hydrothermal extract functions as an AI, and we demonstrate the effect of active MHT components via cell-free and cell-based assays. These results suggest that MHT could be developed for the treatment of hormone-related diseases as well as for the design of AIs.

## Figures and Tables

**Table 1:** Aromatase inhibitory effects of MHT components.

Herbal medicine	Scientific name	Ratio (%) (total 100)	Extraction solvent	IC_50_	Maximum con. (mg/mL)
*Ephedrae Herba*	*Ephedra sinica* Stapf	34.9	H_2_O	1.465 mg/mL	10
*Cinnamomi Ramulus*	*Cinnamomum cassia* Presl	23.3	H_2_O	1.535 mg/mL	10
*Glycyrrhizae Radix et Rhizoma*	*Glycyrrhiza uralensis* Fischer	7.0	H_2_O	1.247 mg/mL	10
*Armeniacae Semen*	*Prunus armeniaca* Linne var. ansu Maximowicz	11.6	H_2_O	>10 mg/mL	10
*Zingiberis Rhizoma Recens*	*Zingiber officinale* Roscoe	11.6	H_2_O	>10 mg/mL	10
*Allii Radix*	*Allium fistulosum* Linne	11.6	H_2_O	>10 mg/mL	10

**Table 2:** Aromatase inhibitory effect of the main compounds from MHT components.

Constituent	Chemical name	Aromatase inhibition (IC_50_)	Maximum con. (mM)
*Ephedrae Herba*	Ephedrine (1)	>10 mM	10

*Cinnamomi Ramulus*	Coumarin (2)	>10 mM	10
	Cinnamic acid (3)	>10 mM	10
	Cinnamaldehyde (4)	>10 mM	10

*Glycyrrhizae Radix et Rhizoma*	Liquiritin (5)	530 *μ*M	10
	Liquiritin apioside (6)	508 *μ*M	10
	Liquiritigenin (7)	1.611 mM	10
	Glycyrrhizin (8)	>10 mM	10

**Table 3:** Aromatase inhibitory effect of MHT and its active compounds in KGN cells.

Name	Aromatase inhibition (IC_50_)	Maximum con.
MHT	246 *μ*g/mL	10 mg/mL
Liquiritin (5)	522 *μ*M	5 mM
Liquiritin apioside (6)	499 *μ*M	5 mM
Liquiritigenin (7)	1.41 mM	5 mM

**Table 4:** Contents of four compounds in MHT (mg/g).

Compound	Contents (*n* = 5)		
	Mean	SD	RSD (%)
1	135.80	0.41	0.30
5	15.58	0.24	1.53
6	19.80	0.25	1.26
7	2.22	0.02	0.85

**Table 5:** Accuracy and intra- and interday precision of the standard compounds in MHT.

Compound	Spiked amount (mg/mL)	Content (mg/mL)		Recovery test (%, *n* = 5)	Precision test (*n* = 5)	
		Expected	Measured		Intraday RSD^*a*^ (%)	Interday RSD (%)
1	0.025	0.0929	0.0929	100.2	0.89	0.29
	0.05	0.1179	0.1192	102.6	0.58	0.59
	0.1	0.1679	0.1708	102.9	0.13	0.25
5	0.025	0.0349	0.0348	99.6	0.83	0.24
	0.05	0.0599	0.0606	101.3	0.34	0.40
	0.1	0.1099	0.1117	101.8	0.06	0.18
6	0.025	0.0328	0.0322	97.8	0.68	0.39
	0.05	0.0578	0.0582	100.7	0.10	0.05
	0.1	0.1078	0.1087	101.0	0.04	0.11
7	0.025	0.0261	0.0267	102.3	2.06	0.18
	0.05	0.0511	0.0523	102.4	0.12	0.10
	0.1	0.1011	0.1029	101.8	0.14	0.07

^a^RSD: relative standard deviation.

**Table 6:** Linearity, LOD, and LOQ of the four standard compounds.

Compounds	*t*_*R*_ (min)	Equation (linear model)^*a*^	Linear range (mg/mL)	*r* ^2*b*^	LOD^*c*^ (*μ*g/mL)	LOQ^*d*^ (*μ*g/mL)
1	9.65	*y* = 10,999,847*x* + 12,569	0.001–0.4	0.9998	0.2507	0.7597
5	14.20	*y* = 15,200,231*x* − 3,254		1.000	0.5774	1.7497
6	14.49	*y* = 18,268,572*x* − 7,852		1.000	0.5259	1.5937
7	18.81	*y* = 28,501,213*x* + 12,569		1.000	0.1714	0.5195

^a^ *y*: peak area at 207 nm; *x*: standard concentration (mg/mL). ^*b*^*r*^2^: coefficient of determination with 11 indicated points on the calibration curves. ^*c*^LOD: limit of detection, S/N = 3.3 (*n* = 7). ^*d*^LOQ: limit of quantification, S/N = 10 (*n* = 7).

## Data Availability

The data used to support the findings of this study are included within the article.
